# Development and Application of High-Internal-Phase Water-in-Oil Emulsions Using Amphiphilic Nanoparticle-Based Emulsifiers

**DOI:** 10.3390/polym16223148

**Published:** 2024-11-12

**Authors:** Chunhua Zhao, Xiujun Wang, Jian Zhang, Yigang Liu, Changlong Liu, Bo Huang, Yang Yang

**Affiliations:** 1 State Key Laboratory of Offshore Oil and Gas Exploitation, Beijing 100027, China; zhaochh16@cnooc.com.cn (C.Z.); wangxj89@cnooc.com.cn (X.W.); zhangjian@cnooc.com.cn (J.Z.); liuyg@cnooc.com.cn (Y.L.); liuchl7@cnooc.com.cn (C.L.); huangbo@cnooc.com.cn (B.H.); 2 CNOOC Research Institute Ltd., Beijing 100027, China; 3 Tianjin Branch of CNOOC Ltd., Tianjin 300452, China; 4 College of Energy, Chengdu University of Technology, Chengdu 610059, China

**Keywords:** W/O emulsions, pH responsiveness, amphiphilicity, nanoparticles

## Abstract

High-internal-phase water-in-oil (W/O) emulsions generated in situ have garnered considerable attention as novel profile control systems. However, conventional emulsifiers are unreactive and poorly dispersed in water, necessitating large dosages and resulting in poor injectivity. In this study, we synthesized amphiphilic nanoparticles (SiO_2_–NH_2_–DAC NPs) containing amine and long-chain alkyl groups using a one-pot method and investigated the stabilized emulsion properties. Our results indicated that W/O emulsions with a water-to-oil ratio (WOR) of 7:3 to 8:2 could be prepared with just 0.1 wt% of SiO_2_–NH_2_–DAC NPs under neutral and basic conditions, with demulsification occurring under acidic conditions (pH = 2.1), demonstrating the pH-responsiveness of the W/O emulsions. The emulsion viscosity increased from 150 to 2555 mPa·s at different WORs. An additional 18.7% oil recovery was achieved using SiO_2_–NH_2_–DAC NPs in a heterogeneous core, highlighting their potential as a promising profile control candidate.

## 1. Introduction

Prolonged water injection in oilfields leads to water channeling, high water content, and severe ineffective circulation [1,2]. In water-swept regions, residual oil saturation is low [3], while unswept areas hold substantial unrecovered crude oil [4,5]. Therefore, profile control techniques are essential to boost the water injection efficiency and enhance oil recovery [6].

Current profile control techniques encompass chemical profile control [7], mechanical profile control, foam profile control [8], microbial profile control [9], and oily sludge profile control [10,11]. Mechanical profile control only addresses water injection issues near the wellbore area [12]. Foam profile control is characterized by high temperature and salt resistance [13], a high resistance coefficient, and a low residual resistance coefficient, but its foaming capacity and stability are significantly influenced by formation conditions [14]. Microbial profile control is highly selective in oil environments but poses a high risk of blockage near the well zone. Chemical profile control allows the selection of polymer gels [15], particles [16,17], microspheres [18], and other systems to suit diverse reservoir conditions [19,20].

The recently developed in situ emulsification profile control technology for crude oil exploits residual oil to create high-internal-phase water-in-oil emulsions (W/O HIPEs) in water-swept areas [21,22,23,24,25], thereby increasing flow resistance and directing subsequent water injections into middle- and low-permeability layers. W/O HIPEs, with oil as the continuous phase and water as the dispersed phase, have an aqueous-phase system fraction exceeding 74% [26]. The emulsion droplets compress and deform, forming a highly viscous fluid [27,28]. Emulsifiers capable of forming W/O emulsions include the Span series [28,29], polyglyceride fatty acid esters, and lecithin [30,31,32,33]. However, these emulsifiers exhibit low hydrophile–lipophile balance values and strong hydrophobicity [34], resulting in their poor dispersion in water. Crude oil often contains acidic components [35,36], which can react with alkalis to form fatty acid soaps at the interface, stabilizing it [37]. However, emulsifiers without reactive groups struggle to synergize with these acidic components, necessitating high emulsifier doses. This challenge complicates demulsification and oil–water separation, increasing the risk of reservoir plugging. Recently, responsive Pickering emulsions have garnered research interest [38,39,40]. Modifying nanoparticles (NPs) with responsive groups can enhance mobility control and facilitate blockage removal via demulsification, particularly under pH-responsiveness. Amines and their derivatives, highly reactive to acids, enable functional NPs to act as pH-responsive switches, altering emulsion types through amine protonation [41].

In this study, we synthesized amine-modified NPs from tetraethyl orthosilicate and 3-aminopropyl triethoxysilane using a one-pot method. Additionally, amphiphilic NPs (SiO_2_–NH_2_–DAC NPs) were created by introducing hydrophobic units via a substitution reaction between acyl chloride and amine groups. These NPs served as emulsifiers to prepare stable W/O emulsions. The basicity of the amine groups on the NP surface enables rapid conversion of the emulsion types and reduces the NP concentration. Our findings suggest that these NPs are highly valuable for enhancing mobility control and demulsification for blockage removal.

## 2. Experimental Section

### 2.1. Materials

Tetraethyl orthosilicate (TEOS, 98%), 3-aminopropyl triethoxysilane (APTES, 99%), dodecanoyl chloride (DAC, 98%), NH_3_·H_2_O (25–28%), NaCl (99.5%), CaCl_2_ (96%), and NaHCO_3_ (99.8%) were purchased from Aladdin Chemical Reagent Company. The parameters of the used crude oil and simulated water are listed in Table 1 and Table 2, respectively.

### 2.2. Synthesis of SiO_2_–NH_2_–DAC NPs

**Scheme 1:**
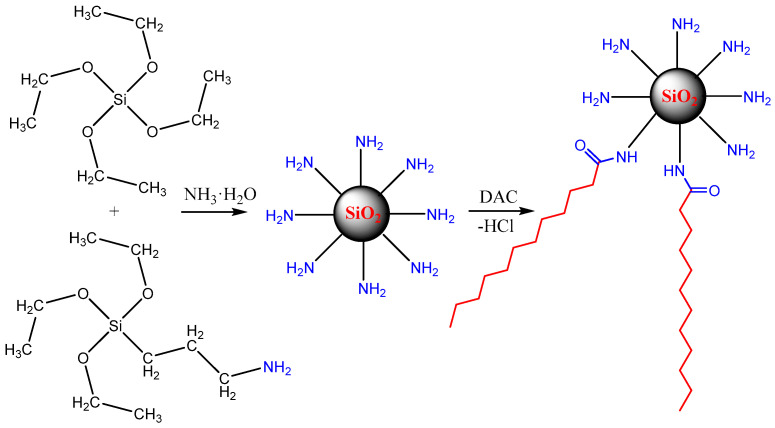
The synthesis route of SiO_2_–NH_2_–DAC NPs.

SiO_2_–NH_2_ NPs were synthesized by a modified Stöber method (Scheme 1). Generally, 2 mL of TEOS was added to a solution containing 40 mL of ethanol, 3 mL of deionized water, and 0.8 mL of NH_3_·H_2_O. The mixture was stirred at 45 °C for 12 h. Afterwards, 2 mL of APTES was added to the mixture to functionalize the surface with amino groups. Then, 0.5 mL of DAC was added dropwise to the mixture at 0 °C. After reaction for 6 h, the mixture was centrifuged and washed with ethanol to obtain a white powder (SiO_2_–NH_2_–DAC NPs).

### 2.3. Structure Characterization

The products were dried in a vacuum oven at 80 °C to constant weight, after mixing a small amount of the samples with KBr, pressing the powder into pellets, and determining the infrared spectrum (Nicolet 6700, Thermo Fisher Scientific, Waltham, MA, USA) in the wavenumber range of 4000–500 cm^−1^. A thermogravimetric analyzer (STA8000, PerkinElmer, Waltham, MA, USA) was used to study the thermal stability of the samples under the test conditions: nitrogen atmosphere, temperature range of 30–800 °C, and heating rate of 10 °C/min. The surface composition of the nanoparticles was determined by X-ray photoelectron spectroscopy (Thermo Kalpha, Thermo Fisher Scientific, USA). The sample was pressed on a glass slide, and the contact angle of the sample was determined by the SL200KS optical contact goniometer at room temperature. The pH of the particle dispersion was adjusted to a specific value at a concentration of 0.01% by mass, and the particle size and zeta potential of the dispersion were determined by a Malvern Zetasizer (Nano ZS90, Malvern, UK). The X-ray diffraction pattern of the sample was determined using a ray diffractometer (D/MAX-RB, Rigaku, Tokyo, Japan) with a scanning angle of 10°–80° and a scanning speed of 10°/min. The dried powder of the sample was dispersed in ethanol and sonicated for 30 min, and then the samples were dropped on a conductive adhesive and their surface morphology was observed by SEM. The dynamic interfacial tension (IFT) between the heavy oil and the different systems was measured by an interfacial tensiometer (SDT, CRUSS, Hamburg, Germany) at 6000 rpm and 90 °C. The IFT value was recorded every 30 s.

### 2.4. Preparation and Evaluation of the Emulsions

The designed amount of SiO_2_–NH_2_–DAC NPs was dispersed in saline water. Then, the crude oil was added to the dispersion. Water-to-oil volume ratios (WORs) of 7:3, 7.5:2.5, and 8:2 were used. The mixture was stirred at 400 r/min for 1 h. An oil bath was used to control the temperature (90 °C). The prepared emulsions were transferred to colorimetric tubes. The tubes were preheated to the specified temperature in an oven. Then, the dewatering volume (V_d_) was recorded at different times. The dewatering rate (R_d_) was calculated to evaluate the stability of the emulsions. V_t_ was the volume of the used dispersion.

$$R_{d}=\frac{V_{d}}{V_{t}}\times 100\%$$

The viscosity of the prepared emulsions was determined using a Brookfield DV2T viscometer (64# spindle, 34 r/min) with a shear rate of 7.34 s^−1^. The rheological curves of the prepared polymer solution were plotted using a rheometer (Malvern Kinexus, Malvern, UK). In rheological experiments, the frequency sweep curve was plotted at frequencies ranging from 0.1 to 10 Hz using a rheometer (MCR 302, Anton Paar, Graz, Austria) and CP50-1-SN30644 (Anton Paar, Austria) plate clamps (diameter = 0.099 mm). The micrographs of the prepared emulsions were measured by an optical microscope (CX40, Sunny Optical Technology Company, Ningbo, China) at room temperature.

### 2.5. Oil Displacement Ability in Heterogeneous Core

**Figure 1:**
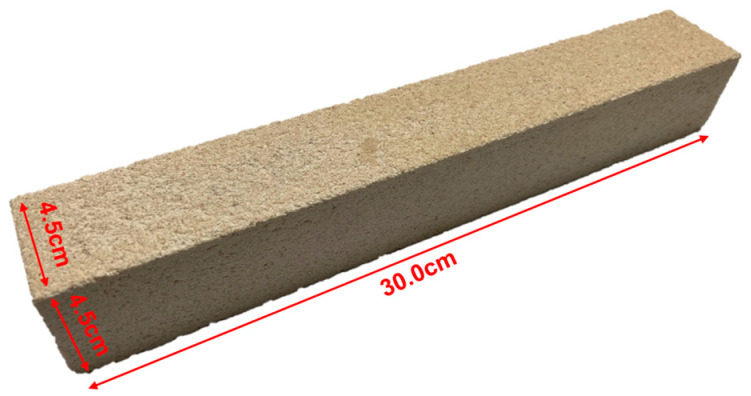
Heterogeneous core used in core flooding experiment.

A two-layer heterogeneous sandstone core (permeability 200/600 mD, determined by perm-plug method; Figure 1) with a cuboid shape (4.5 cm × 4.5 cm × 30 cm) was saturated with crude oil (porosity 23.4%, oil saturation 66.5%). The core was placed in the core holder with the casing pressure at 30 °C. Then, water flooding was conducted at a constant injection flow rate of 0.3 mL/min. After that, a 0.3 PV nanoparticle slug was injected when the water cut reached 80%. Finally, water flooding was conducted continually until the economic limit (98%) was reached. The pressure and enhanced oil recovery (EOR) value were recorded.

## 3. Results and Discussion

### 3.1. Results of Structural Characterization

**Figure 2:**
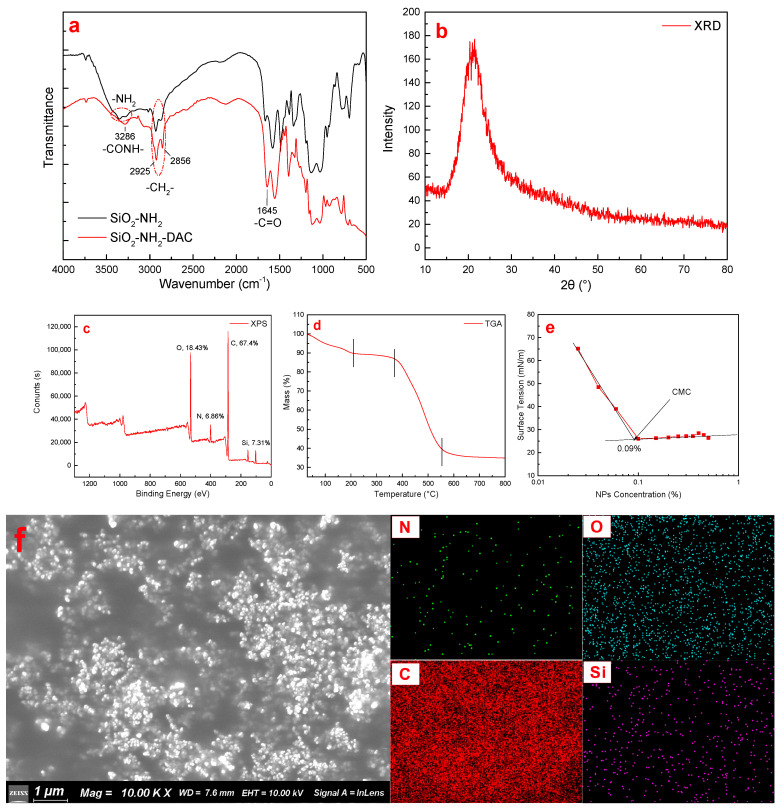
The structural characterization of SiO_2_–NH_2_–DAC: (**a**) FTIR spectra, (**b**) XRD spectrum, (**c**) XPS spectrum, (**d**) TGA curve, (**e**) surface tension curve, (**f**) SEM and mapping images.

In Figure 2a, the peak at 3330–3359 cm^–1^ corresponds to the –NH_2_ group, while the peaks at 2856 and 2925 cm^–1^ correspond to the –CH_2_– group. The –OH peak of silica disappeared, confirming an adequate reaction between silica and the amine group. Additionally, in the SiO_2_–NH_2_–DAC spectrum, the N–H stretching peak in the amide group appeared at 3286 cm^–1^, and the –C=O vibration peak appeared at 1645 cm^–1^, indicating that DAC reacts with the amine group to form an amide bond. These results confirm the successful introduction of amine groups and hydrophobic units onto the silica surface.

The X-ray diffraction patterns (Figure 2b) displayed a characteristic peak at 21.5°, confirming the product as amorphous silica. X-ray photoelectron spectroscopy (Figure 2c) revealed the presence of silicon (7.31%), carbon (67.4%), nitrogen (6.86%), and oxygen (18.43%). The thermogravimetric analysis (Figure 2d) indicated weight loss below 200 °C, owing to crystalline water in the amphiphilic NPs, and between 380 and 550 °C, caused by the decomposition of the organic carbon chain. Beyond 800 °C, the weight stabilized at 35%, indicating significant organic content on the NPs’ surface. The critical micelle concentration (CMC) was about 0.09% (Figure 2e). Scanning electron microscopy (Figure 2f) showed that the SiO_2_–NH_2_–DAC NPs were spherical, averaging 100 nm in size.

### 3.2. Emulsifying Capacity of SiO_2_–NH_2_–DAC NPs

**Figure 3:**
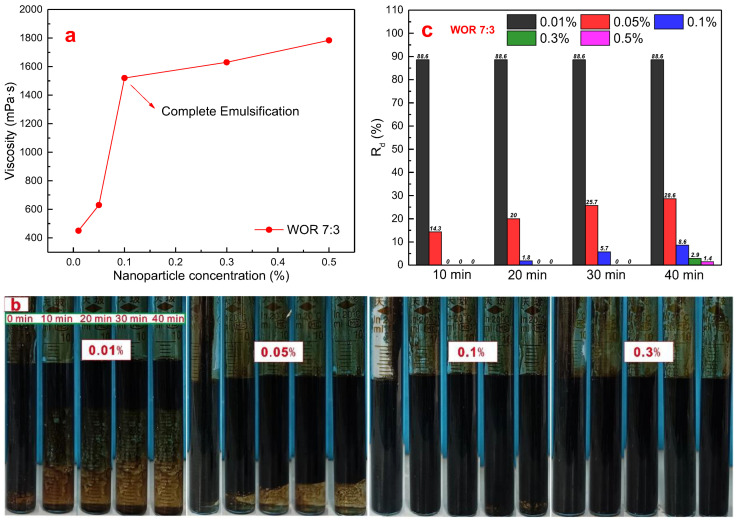
The emulsion properties of SiO_2_–NH_2_–DAC NPs at different concentrations: (**a**) the influence of nanoparticle concentration on the viscosity of the emulsions; (**b**) the photographs of the emulsions; (**c**) the dewatering rate at different times.

The surface of SiO_2_–NH_2_–DAC NPs features amine groups and long carbon chains, making it amphiphilic and aiding in emulsion formation. At high WOR, emulsion droplets are compressed and deformed, creating a high-viscosity fluid. As shown in Figure 3a, the viscosity sharply increased to 1520 mPa·s at an NP concentration of 0.1%, significantly higher than the crude oil’s viscosity, indicating thorough emulsification and suggesting effective mobility control; this result is corroborated by the photographs shown in Figure 3b. NPs spontaneously aggregate at the two-phase interface, forming an interfacial membrane [42]. The membrane’s strength increases with increasing NP concentration; thus, when the NP concentration exceeds 0.1%, the emulsion viscosity continues to increase. The interfacial membrane also prevents emulsion droplet coalescence, stabilizing the emulsion [43,44]. Consequently, the R_d_ value decreased markedly, showing good emulsion stability (Figure 3c).

**Figure 4:**
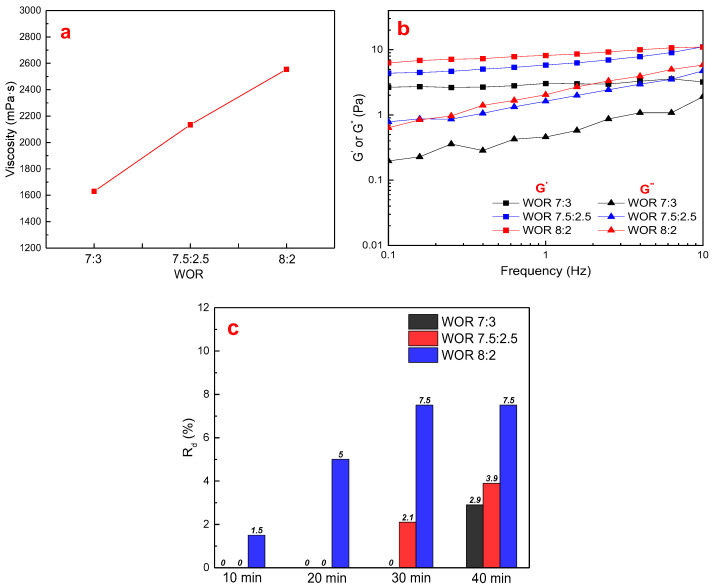
The emulsion properties of SiO_2_–NH_2_–DAC NPs (0.3%) at different WORs: (**a**) the influence of WOR on the viscosity of the emulsions; (**b**) the viscoelasticity of the emulsions; (**c**) the dewatering rate at different times.

During water flooding, the water–oil ratio (WOR) increases in water-swept areas, particularly in medium- and high-permeability layers, reducing residual oil saturation and flow resistance, and causing an ineffective water injection cycle. In this study, three WORs were used. Figure 4a,b reveal that as the WOR increased, the viscosity and viscous modulus increased rapidly owing to the compression and deformation of emulsion droplets. Additionally, NPs aggregated at the oil–water interface, forming an elastic interface membrane, making the elastic modulus significantly higher than the viscous modulus. However, the emulsion stability decreased with increasing R_d_ value at a high WOR (Figure 4c). Studies suggest that NPs can stabilize emulsions with surfactants [45,46,47]. Thus, to increase the WOR, the NP dosage can be increased, or a small amount of emulsifier can be added.

### 3.3. pH Sensitivity of SiO_2_–NH_2_–DAC NPs

**Scheme 2:**
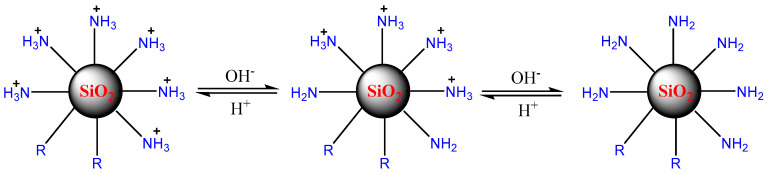
The pH-responsiveness of SiO_2_–NH_2_–DAC NPs.

The SiO_2_–NH_2_–DAC NPs’ surface contains two characteristic units: an amine group and a long-chain alkyl group. The amine group imparts pH-responsiveness to the NPs (Scheme 2).

**Figure 5:**
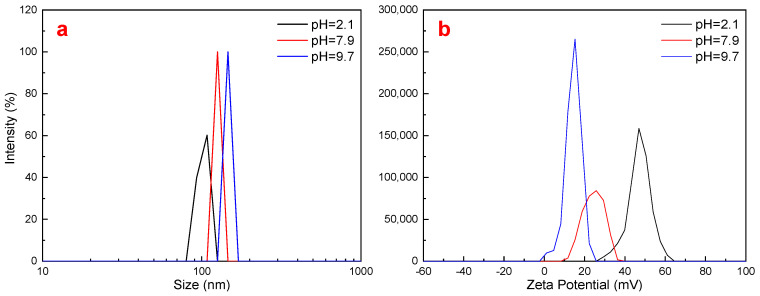
The influence of pH on the (**a**) size and (**b**) zeta potential of SiO_2_–NH_2_–DAC.

Upon protonating the amine group, the NPs showed increased surface charge and a significantly elevated positive zeta potential (Table 3 and Figure 5). This electrostatic repulsion reduced the binding forces between NPs, enhancing their dispersity and decreasing their size to 29.9 nm under acidic conditions. As the pH increased, protonation decreased, leading to a lower zeta potential. Consequently, the repulsive forces weakened, causing an increase in the NPs’ size. These findings demonstrate a positive correlation between zeta potential and NP dispersibility, with a higher zeta potential leading to better dispersibility.

**Figure 6:**
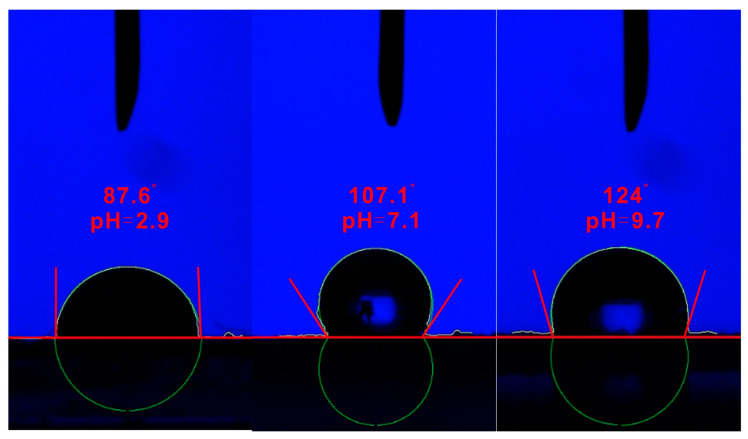
The contact angle at different pH.

The pH-responsiveness of SiO_2_–NH_2_–DAC NPs is evident from the contact angle measurements. As shown in Figure 6, under alkaline conditions, the weak hydrophilicity of the amine groups and strong hydrophobicity of the alkyl chains yielded a contact angle of 124°. As the pH decreased, the amine groups converted into hydrophilic amine salts, reducing the contact angle to 107.1° and 87.6° at pH = 7.1 and 2.9, respectively.

**Figure 7:**
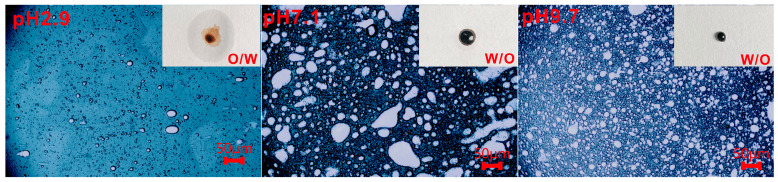
The micrographs of the emulsions at different pH.

NPs with amphiphilic properties, such as surfactants, rapidly aggregate at the two-phase interface, thereby stabilizing it. Hydrophobic emulsifiers are used for W/O emulsions, while hydrophilic emulsifiers prepare oil-in-water (O/W) emulsions [48]. Utilizing the pH-responsiveness of SiO_2_–NH_2_–DAC NPs, we aimed to control emulsion types by adjusting the amine group’s protonation state. As illustrated in Figure 7, the NPs were hydrophobic, forming a W/O emulsion, but under acidic conditions their hydrophilicity increased, forming an O/W emulsion. This change in emulsion type can enhance mobility control and blockage removal.

**Figure 8:**
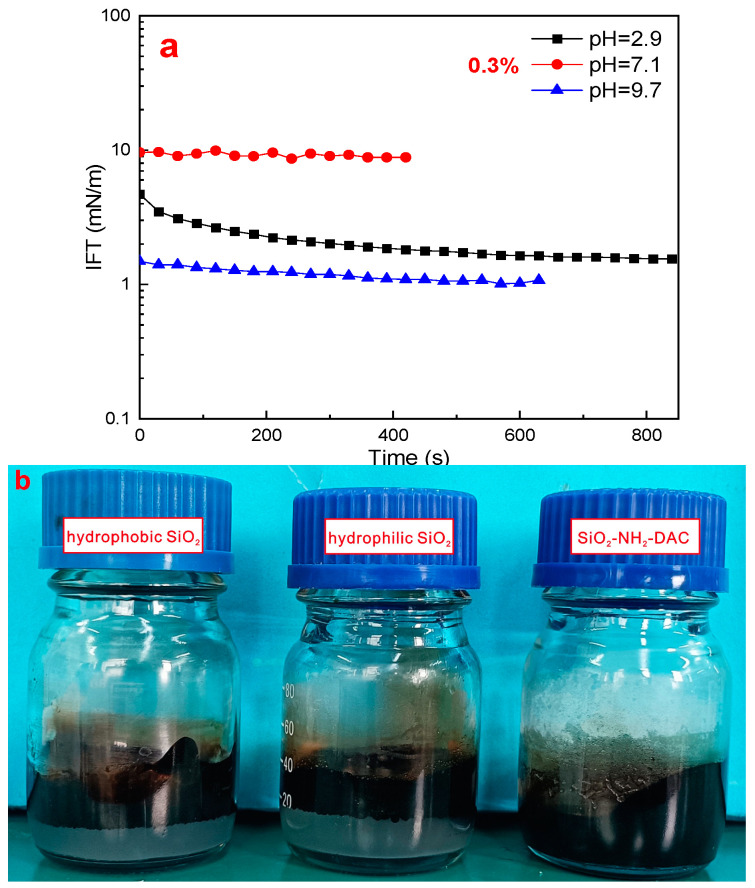
(**a**) The influence of pH on the IFT, and (**b**) the emulsion photographs of different nanoparticles.

The amine group, an alkaline functional group, reacts with the acidic components in crude oil to form fatty acid soaps at the two-phase interface, thereby stabilizing it. Figure 8a shows that interfacial tension (IFT) is strongly influenced by the protonation of the amine group on NP surfaces. The amine group’s ability to generate surfactants in situ is crucial for SiO_2_–NH_2_–DAC to stabilize emulsions at low concentrations. In contrast, stable W/O emulsions cannot be created using hydrophobic or hydrophilic SiO_2_ (Figure 8b). Increased protonation of the amine group reduces its reactivity with acidic components, increasing the interfacial tension under neutral conditions. Full protonation forms an amine salt with a long-chain alkyl group, producing a giant cationic surfactant that effectively reduces interfacial tension.

### 3.4. Core Flooding Experiments of SiO_2_–NH_2_–DAC NPs

**Figure 9:**
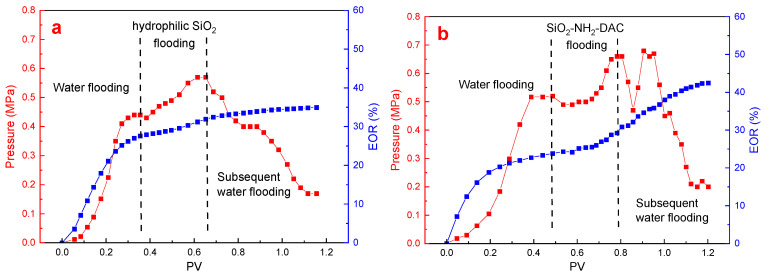
The displacement results of hydrophilic SiO_2_ (**a**) and SiO_2_–NH_2_–DAC NPs (**b**).

Due to the core’s heterogeneity, only 23.8% oil recovery was achieved by the end of water flooding (Figure 9). Injection of 0.3 PV SiO_2_–NH_2_–DAC NPs gradually increased the injection pressure to 0.66 MPa, with notable fluctuations in the subsequent water flooding stage. This pressure increase can be attributed to an increase in the displacement resistance caused by the in situ HIPE formation. Unlike polymer gels, emulsion drops are flexible, leading to dynamic capture and re-migration characteristics that cause pressure fluctuations. As HIPE formation is time-consuming, the pressure increase was delayed until after the injection of 0.2 PV SiO_2_–NH_2_–DAC NPs. The oil recovery improved owing to the profile control effect of the HIPEs formed in situ, which enhanced the sweep efficiency by migrating the flooding phase into the core’s low-permeability areas. Consequently, oil recovery rose to 42.5% after subsequent water flooding, achieving an additional 18.7% oil recovery with SiO_2_–NH_2_–DAC NPs. In comparison, the enhanced oil recovery of hydrophilic SiO_2_ was only 7.3%, which is much lower than that of the SiO_2_–NH_2_–DAC NPs. The results demonstrate the potential of SiO_2_–NH_2_–DAC NPs as a profile control agent for enhanced oil recovery.

## 4. Conclusions

In this study, amine-modified NPs were synthesized from tetraethyl orthosilicate and 3-aminopropyl triethoxysilane using a one-pot method, and amphiphilic NPs (SiO_2_–NH_2_–DAC NPs) were prepared by introducing hydrophobic units via a substitution reaction between acyl chloride and amine groups. SiO_2_–NH_2_–DAC NPs, featuring amine and long-chain alkyl groups with amphiphilic properties, served as emulsifiers for W/O HIPEs up to a WOR of 8:2. The pH-responsive amine groups altered the contact angle from hydrophobic to hydrophilic, converting the emulsion type from W/O to O/W. These alkaline amine groups also reacted with acidic crude oil components, reducing the NP dosage. A concentration of 0.1% was found to be sufficient to form HIPEs effectively. HIPE formation in the core was validated by the observed increase and fluctuations in pressure during displacement. The SiO_2_–NH_2_–DAC NPs achieved an additional 18.7% oil recovery, demonstrating significant potential for profile control.

## Figures and Tables

**Table 1:** The parameters of the used crude oil.

Density (g/cm^3^)	Acid Value (mg KOH/g)	Viscosity (mPa·s)
0.917	3.1	151

**Table 2:** The parameters of the simulated water.

Na^+^ (mg/L)	K^+^ (mg/L)	Ca^2+^ (mg/L)	Mg^2+^ (mg/L)	Cl^−^ (mg/L)	HCO_3_^−^ (mg/L)	SO_4_^2−^ (mg/L)	Salinity (mg/L)
10,052.08	105.96	366.88	1172.08	17,588.09	153.25	2691	32,129.33

**Table 3:** The size and zeta potential of SiO_2_–NH_2_–DAC at different pH.

pH	2.9	7.1	9.7
Average Size (nm)	29.9	125.6	146.1
Zeta Potential (mV)	47.21	23.01	12.43

## Data Availability

The original contributions presented in this study are included in the article; further inquiries can be directed to the corresponding author.
